# Prescribing trends and time series analysis of blood pressure-lowering drugs among patients with dementia: a multinational database study

**DOI:** 10.1016/j.eclinm.2025.103595

**Published:** 2025-10-30

**Authors:** Edmund C.L. Cheung, Yunzhang Wang, Lisa Kalisch Ellett, Matthew Adesuyan, Máté Szilcz, Sonia Shah, Nicole Pratt, Ruth Brauer, Yogini H. Jani, Robert D. Smith, Hao Luo, Jacqueline K. Yuen, Sara Hägg, Celine S.L. Chui

**Affiliations:** a Centre for Safe Medication Practice and Research, Department of Pharmacology and Pharmacy, Li Ka Shing Faculty of Medicine, The University of Hong Kong, Hong Kong SAR, China; b Department of Medical Epidemiology and Biostatistics, Karolinska Institutet, Stockholm, Sweden; c Quality Use of Medicines and Pharmacy Research Centre, Clinical and Health Sciences, University of South Australia, Adelaide, SA, Australia; d Research Department of Practice and Policy, UCL School of Pharmacy, London, UK; e Centre for Medicines Optimisation Research and Education, University College London Hospitals NHS Foundation Trust, London, UK; f Institute for Molecular Bioscience, The University of Queensland, Brisbane, QLD, Australia; g Department of Public Health and Medicinal Administration, Faculty of Health Sciences, University of Macau, Macau SAR, China; h School of Public Health Sciences, University of Waterloo, Waterloo, Ontario, Canada; i Department of Social Work and Social Administration, The University of Hong Kong, Hong Kong SAR, China; j Department of Medicine, School of Clinical Medicine, Li Ka Shing Faculty of Medicine, The University of Hong Kong, Hong Kong SAR, China; k School of Public Health, Li Ka Shing Faculty of Medicine, The University of Hong Kong, Hong Kong SAR, China; l School of Nursing, Li Ka Shing Faculty of Medicine, The University of Hong Kong, Hong Kong SAR, China; m Advance Data Analytics for Medical Science (ADAMS) Limited, Hong Kong SAR, China

**Keywords:** Dementia, Blood pressure-lowering drugs, Antihypertensive drugs, Hypertension, Prescribing trends

## Abstract

**Background:**

Hypertension is common among people living with dementia and blood pressure-lowering drug (BPLD) treatment in dementia patients may vary widely between individuals and countries/regions. This study aimed to describe and evaluate how prescribing trends of BPLD change before and after incident dementia diagnosis using electronic health record databases across four countries/regions.

**Methods:**

Electronic health records were collected from Hong Kong, United Kingdom, Sweden, and Australia for this study. Study dates were based on data availability from each database and ranged from January 1st, 2000 to December 31st, 2020. The target population were people diagnosed with dementia, with a prior diagnosis of hypertension and prescription of BPLD. Time series analysis, similar to interrupted time series, was conducted to evaluate the prescribing trends of BPLD three years before and after dementia diagnosis. The primary outcome of interest was the monthly proportion of patients prescribed with a BPLD.

**Findings:**

31,873 patients from Hong Kong, 59,108 from the UK, 5034 from Sweden and 12,807 from Australia were included in this study. The mean age ranged from 78.3 years in Australia to 81.1 years in Hong Kong at time series entry. BPLD prescribing decreased in the three years before dementia diagnosis (negative pre-dementia slope) across all four databases. A significant immediate increase in antihypertensive prescribing was observed immediately after incident dementia diagnosis in Hong Kong (Estimate∗100: 4.66, 95% CI: 3.79–5.53), Sweden (Estimate∗100: 2.24, 95% CI: 1.60–2.88), and Australia (Estimate∗100: 1.58, 95% CI: 1.07–2.09), while there was no significant level change in the UK. BPLD prescribing declined in the three years after diagnosis for all databases.

**Interpretation:**

This is the largest multinational population-based study to date investigating prescribing trends of BPLD before and after dementia diagnosis. The time series analysis results suggested that there was increased vigilance of blood pressure control shortly after dementia diagnosis.

**Funding:**

This study was supported by the Hong Kong Research Grants Council General Research Fund, No. 17113720.

**Research in context**

**Evidence before this study**

We searched PubMed for studies published from database inception to May 9, 2025, using the search terms: (“blood pressure-lowering drug∗” OR “antihypertensive∗” OR (“hypertension” AND “treatment”)) AND (“dementia” OR “Alzheimer's disease” OR “vascular dementia”), which yielded 1395 articles. After excluding articles deemed not to be relevant based on their article titles, we identified and reviewed abstracts of articles including studies of blood pressure-lowering drug (BPLD) prescribing in people with dementia and other articles describing hypertension treatment in dementia or older adults. The management of hypertension in people with dementia is under-examined and optimal treatment may differ not only between individuals but also between different populations. Previous studies of BPLD prescribing in people with dementia have also been limited in study sample size and population diversity.

**Added value of this study**

To our knowledge, this is the largest multinational population-based study to date investigating prescribing trends of BPLD before and after dementia diagnosis using electronic health databases across four countries/regions including Hong Kong, United Kingdom, Sweden, and Australia. The findings of this study add to the available evidence to describe how hypertension is treated around the time of a patient's incident dementia diagnosis. As a common protocol was used to harmonise the study approach and design, the trends can be compared between countries/regions to show how prescribing practices differ between different populations. The time series analysis findings suggested that attention to hypertension management was heightened immediately after dementia diagnosis in general.

**Implications of all the available evidence**

BPLD prescribing trends differ between different countries/regions and can change before and after incident dementia diagnosis. Both clinicians and carers of patients with dementia should assess healthcare priorities and treatment goals when prescribing BPLD. Further research is recommended to evaluate associations between different management strategies of hypertension and health outcomes in people with dementia to inform best practice hypertension care.

## Introduction

Ageing is a major public health issue and the most important risk factor for developing morbidities such as hypertension and dementia. Developing hypertension in midlife and hypotension in late-life adulthood is also associated with an increased risk of incident dementia,^1^, ^2^, ^3^, ^4^ while blood pressure changes after dementia diagnosis are complex and can vary.^5^ The relationship between neurodegenerative diseases including dementia and blood pressure is bidirectional, as hypertension can promote neurodegeneration while neurodegenerative diseases can disrupt blood pressure regulation and potentially exacerbate disease progression.^6^^,^^7^ Understanding this interplay is important for developing effective strategies for prevention and treatment of both hypertension and dementia.

Poor blood pressure control not only increases the risk of dementia but may also worsen cognitive decline in people with dementia. Although the risk of dementia following blood pressure-lowering treatment has been extensively studied,^8^^,^^9^ prescribing behaviour around the time of a patient's dementia diagnosis has not been thoroughly investigated and such research can provide insights on how hypertension is managed in people living with dementia. Best practice management of hypertension in people with dementia is also not well described and treatment guidelines do not tend to give recommendations on hypertension treatment for people with dementia. With the differing degrees of burdens from concurrent conditions and frailty often seen in older patients, hypertension treatment after dementia diagnosis may differ not only between individuals but also between ethnic populations in different countries.^10^, ^11^, ^12^

Previous studies have shown that patterns of medicine or healthcare utilization can change following dementia diagnosis. For example, studies in the US showed that the use of cardiometabolic medications declined after incident dementia diagnosis,^13^^,^^14^ while healthcare trajectories can be greatly affected by multiple factors including sex, comorbidities and living situation.^15^^,^^16^ The clinical questions of interest regarding blood pressure-lowering drugs (BPLD) include how and why prescribing patterns change before and after incident dementia diagnosis. Previous studies of antihypertensive prescribing trends in people with dementia have been restricted in terms of study sample size and setting.^17^^,^^18^ There is a need for a large-scale study to investigate these prescribing trends in ethnically diverse populations from different countries to observe the current practice of antihypertensive therapy as understanding such patterns is an important step towards improving the management of hypertension in people with dementia.

This study aims to describe regional differences in prescribing of BPLD/antihypertensives (hereby referred interchangeably) in patients across Asia, Australia, and Europe, and to evaluate whether there are changes in prescribing trends before and after incident dementia diagnosis in patients with hypertension.

## Methods

This is a population-based retrospective multinational database study, using electronic medical records from Hong Kong, United Kingdom, Sweden and Australia. A common protocol approach was used to harmonise study design, analysis specifics and results format across the databases. This protocol was developed between each collaborating site and included instructions to conduct the study, data shells for analysis, and result templates to follow. All raw data was retrieved and analysed within each collaborating institute without needing to transfer confidential patient data of individuals. This harmonisation approach has been used in previous studies to put together results using multiple databases from different countries/regions.^19^, ^20^, ^21^

### Data sources

Electronic health records including demographics, diagnosis records, prescription records and death dates were retrieved from the four databases: Clinical Data Analysis and Reporting System (Hong Kong), IQVIA Medical Research Database (United Kingdom), the Swedish Prescribed Drug Register and the Swedish Dementia Registry (Sweden), and Pharmaceutical Benefits Scheme 10% sample data collection (Australia). These databases have been validated and have been used extensively in previous studies, described in detail in terms of data capture and representativeness in Supplementary Material 1 and 2. The study dates that databases were analysed were based on data availability and ranged from January 1st, 2000 to December 31st, 2020 across databases. All collaborating sites obtained ethical and/or governance approval prior to data retrieval for this study.

### Study design

This is a descriptive study of prescribing trends over a time series with an analytical component to support interpretation. The impact of incident dementia diagnosis on BPLD prescribing was evaluated using a time series analysis to compare the prescribing trends three years before and up to three years in the available time after a patients’ incident dementia diagnosis. The three-year observation window was set based on the median survival time being approximately three years after incident dementia diagnosis in older adults,^22^ as well as the time to diagnosis in dementia from first symptoms being 3.5 years,^23^ making it a clinically relevant window for prescribing changes. Most prescriptions also have durations of around one month per script or up to six months maximum in the countries/regions included. Any manifestation of cognitive impairment is likely to be detected by clinicians within three years to make corresponding changes in prescribing. This time series method is similar to interrupted time series analysis used to evaluate the impact of population-level policy changes or quality improvement programmes on healthcare outcomes using longitudinal data measured at multiple timepoints before and after an intervention.^24^^,^^25^ In this study, incident dementia diagnosis was used as the breakpoint in place of the common policy intervention in an interrupted time series analysis. The definition of incident dementia diagnosis for each database is described in Supplementary Material 2.

#### Study population

**Fig. 1:**
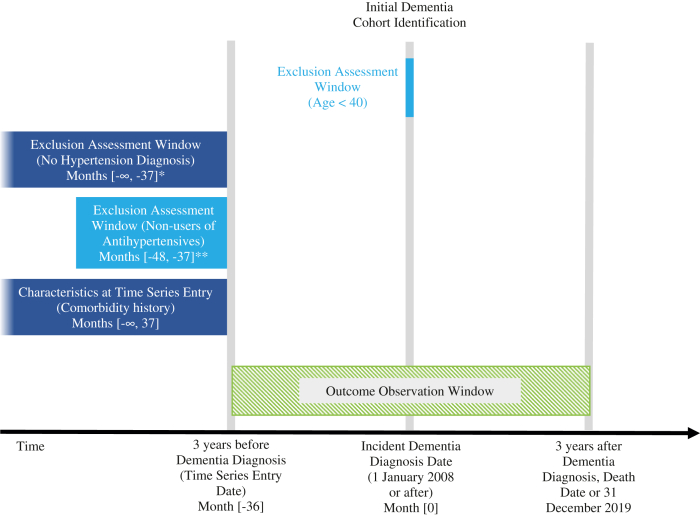
**Cohort identification diagram: Patients with dementia, diagnosed with hypertension and requiring antihypertensive treatment at least 3 years before dementia diagnosis**. ∗Hypertension diagnosis looking back from 3 years before dementia to the earliest available diagnosis data for each database. ∗∗Antihypertensive prescription in a 1-year baseline period prior to 3 years before dementia or looking back to the earliest prescription data for each database.

The target population was patients aged 40 years or older diagnosed with dementia, with a prior diagnosis of hypertension at least three years before their first diagnosis of dementia. In the Australian database, the first dispensing of BPLD was used as a proxy for diagnosis of hypertension using a validated mapping index^26^ as diagnosis data were not available. The baseline period was set looking back from the three-year timepoint before incident dementia diagnosis to ensure a consistent study population before entering the time series. Patients also required a prescription of any BPLD in the year before entering the time series. BPLD classes included angiotensin-converting enzyme inhibitors, angiotensin II receptor blockers, calcium channel blockers, diuretics, beta-blockers, vasodilators, centrally acting alpha-2 agonists, and alpha-1 blockers. Details of identification of the study population are presented in Fig. 1. Medical codes used to identify dementia and hypertension diagnosis are described in Supplementary Material 3 while codes used to identify BPLD dispensing are described in Supplementary Material 4.

#### Outcomes of interest

The primary outcome of interest was the monthly proportion of patients prescribed or dispensed a BPLD, with secondary outcomes of the monthly proportion of patients with uncontrolled blood pressure measurements (UBP) and the monthly proportion of patients who had records of healthcare resource utilization (HCRU) to support the interpretation of the primary outcome. Some databases were unable to calculate certain secondary outcomes depending on data availability.

i) Blood pressure-lowering drug (BPLD): patients were counted if they received a prescription within each month. For prescriptions spanning continuously over several months, they were counted in each month. Data for this outcome were available in all datasets.
ii) Uncontrolled blood pressure (UBP): high blood pressure was defined as 140/90 mmHg or over (≥140/90, aged under 80) or 150/90 mmHg or over (≥150/90, aged 80 or over).^27^ Low blood pressure was defined as under 90/60 mmHg (<90/60).^28^ Patients were defined as uncontrolled if they had a measurement of both systolic blood pressure (SBP) and diastolic blood pressure (DBP) crossing the threshold stated above of either high blood pressure or low blood pressure each month. If a patient had records of multiple blood pressure measurements in a month, the lowest blood pressure measurement was used, defined as the SBP and DBP pair with the lowest mean between the SBP and DBP value. Data for this outcome were only available in Hong Kong and the UK.
iii) Healthcare resource utilization (HCRU): patients were counted if they had a record of any healthcare visit (outpatient and/or inpatient) within each month. For inpatient hospitalisation visits spanning continuously over several months, they were counted in each month. Data for this outcome were only available in Hong Kong and Sweden.

### Statistical analysis

For the time series analysis, a segmented autoregressive error model was used to compare the monthly proportion of patients prescribed BPLD and other outcomes (Supplementary Material 5).^29^^,^^30^ A three-month period before, on, and after dementia diagnosis month was removed from the analysis, as this was the common period where all patients were highly likely to have been exposed to healthcare services, which may have overestimated the results. All outcomes were calculated monthly relative to patients’ incident dementia diagnosis date. For each monthly timepoint, patients were only considered in the denominator of the proportion from study entry up to death, loss to follow-up or end of data collection, whichever occurred first. In this way, patients were counted in the denominator up to their month of death/loss to follow-up/end of data collection and excluded from subsequent months. In the Australian database, only the year of death was available without exact dates, therefore, people who had a year of death recorded were excluded from all trends from January of their year of death onwards. The pre-dementia slope (trend before dementia), level change (immediate effect following dementia) and slope change (gradual effect following dementia) estimates from the time series analysis were presented with 95% confidence intervals (95% CI) for each outcome for each database. A p-value of 0.05 or lower was considered statistically significant. All analyses were conducted in R version 4.3.0 (R Foundation for Statistical Computing, Vienna, Austria), Stata 17 (StataCorp, College Station, TX) and Statistical Analysis System (SAS) v9.4 (SAS Institute, Cary, NC).

### Ethics statement

Hong Kong: This study was approved by the Institutional Review Board of the University of Hong Kong/Hospital Authority West Cluster (Reference Number: UW 20-053).

United Kingdom: This study was approved by the IMRD-UK Scientific Review Committee in March 2022 (Reference Number: 20SRC073-A1).

Sweden: This study was approved by the Regional Ethical Review Board of Stockholm (dnr: 2016/1001-31/4, 2020-03525; 2021-02004).

Australia: The study was approved by the Services Australia External Request Evaluation Committee (EREC approval RMS2351).

All clinical data was anonymised and informed consent was not required under the regulations of each study site in this multinational database study.

### Role of the funding source

This study was supported by the Hong Kong Research Grants Council General Research Fund, No. 17113720. The study sponsor was not involved in study design; in the collection, analysis, and interpretation of data; in the writing of the report; and in the decision to submit the paper for publication.

## Results

A total of 31,873 patients from Hong Kong, 59,108 from the UK, 5034 from Sweden and 12,807 from Australia were included in this study. At time series entry three years before incident dementia diagnosis, the mean age was similar across databases, ranged from the lowest in Australia (78.3) to the highest in Hong Kong (81.3). Baseline characteristics are presented in Table 1.

**Table 1:** Baseline characteristics of cohorts in each database—three years before dementia diagnosis.

Baseline characteristics	Hong Kong N = 31,873	United Kingdom N = 59,108	Sweden N = 5034	Australia N = 12,807
**Age (mean, SD)**	81.1 (7.8)	79.5 (7.1)	78.7 (6.6)	78.3 (9.9)
**Age group (N, %)**	–	–	–	–
≤64	990 (3.1)	1989 (3.4)	149 (3.0)	828 (6.5)
65–74	4736 (14.9)	12,251 (20.7)	1081 (21.5)	2785 (21.7)
75–84	15,171 (47.6)	31,231 (52.8)	2871 (57.0)	5930 (46.3)
≥85	10,976 (34.4)	13,637 (23.1)	933 (18.5)	3264 (25.5)
**Female (N, %)**	19,833 (62.2)	38,384 (64.9)	2619 (52.0)	7219 (56.4)
**Dementia subtype (N, %)**^b^	–		–	–
Alzheimer's disease	4052 (12.7)	20,913 (35.4)	952 (18.9)	–
Vascular dementia	5376 (16.9)	16,914 (28.6)	1471 (29.3)	–
Unspecified dementia	21,818 (68.5)	19,223 (32.5)	1207 (24.0)	–
Other causes	627 (2.0)	808 (1.4)	111 (2.2)	–
Frontotemporal dementia	–	49 (0.1)	37 (0.7)	–
Dementia with Lewy bodies	–	1201 (2.0)	97 (1.9)	–
Mixed Alzheimer's disease and Vascular dementia	–	–	1088 (18.0)	–
**Comorbidity history (N, %)**	–	–	–	–
Myocardial infarction	1828 (5.7)	5807 (9.8)	685 (13.6)	–
Congestive heart failure	4532 (14.2)	4631 (7.8)	820 (16.3)	1191 (9.3)
Peripheral vascular disease	2210 (6.9)	2959 (5.0)	543 (10.8)	–
Cerebrovascular disease	10,618 (33.3)	10,790 (18.3)	828 (16.4)	–
Chronic pulmonary disease	3390 (10.6)	12,515 (21.2)	391 (7.8)	1922 (15.0)
Connective tissue disease	268 (0.8)	4186 (7.1)	333 (6.6)	–
Ulcer disease	3291 (10.3)	3834 (6.5)	127 (2.5)	4496 (35.1)
Liver disease	502 (1.6)	950 (1.6)	47 (0.9)	119 (0.9)
Diabetes	11,615 (36.4)	13,371 (22.6)	1710 (34.0)	2075 (16.2)
Hemiplegia/paraplegia	2414 (7.6)	634 (1.1)	24 (0.5)	
Renal disease	2739 (8.6)	16,710 (28.3)	280 (5.6)	54 (0,42)
Any tumour	2447 (7.7)	12,518 (21.2)	724 (14.4)	70 (0.55)
Leukaemia	41 (0.1)	28 (0.1)	36 (0.7)	–
Lymphoma	67 (0.2)	12 (0.0)	37 (0.7)	–
AIDS	a	a	–	–
**Charlson Comorbidity Index**^b^**—Hong Kong, United Kingdom, Sweden (mean, SD)**	5.30 (1.78)	6.68 (1.98)	5.27 (1.74)	–
**RxRisk comorbidities—Australia (mean, SD)**	–	–	–	4.0 (3.11)

a Patient count less than 10.

b Calculation of Charlson Comorbidity Index described in Supplementary Material 6.

### Primary outcome: BPLD

**Fig. 2:**
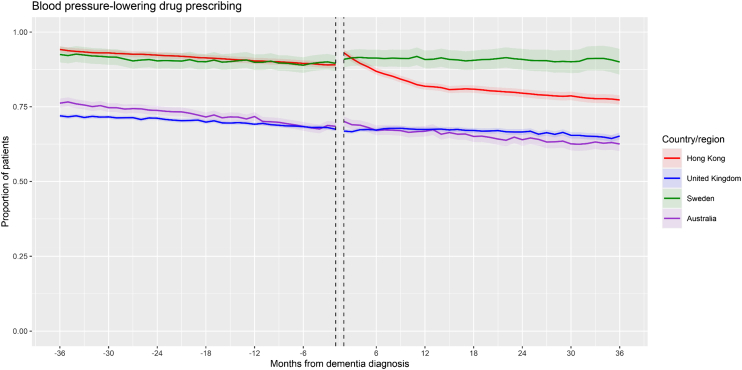
**Time series plot (All databases)—Blood pressure-lowering drug prescribing**. Note: Shaded areas represent 95% confidence intervals of monthly proportions.

The monthly proportion plot of the time series for BPLD for each database is presented in Fig. 2, while the time series estimates are presented in Table 2. In each database, a significant negative pre-dementia slope for BPLD was observed indicating a decreasing trend in antihypertensive prescribing prior to dementia diagnosis. A significant increase in the level change (immediate increase in antihypertensive prescribing) was observed immediately after incident dementia diagnosis in Hong Kong (Estimate∗100: 4.66, 95% CI: 3.79–5.53), Sweden (Estimate∗100: 2.24, 95% CI: 1.60–2.88), and Australia (Estimate∗100: 1.58, 95% CI: 1.07–2.09), while there was no significant level change in the UK. The slope was significantly decreased (greater decreasing trend) in Hong Kong after dementia diagnosis. In the other databases, the slope was increased but continued to be negative (lesser decreasing trend) after dementia diagnosis, and this slope change was significant in Sweden and Australia, whereas it was non-significant in the UK.

**Table 2:** Time series analysis estimates.

Outcome	Hong Kong			United Kingdom			Sweden			Australia		
	Estimate ∗100	95% CI	p-value	Estimate ∗100	95% CI	p-value	Estimate ∗100	95% CI	p-value	Estimate ∗100	95% CI	p-value
**All blood pressure-lowering drugs**												
Pre-dementia slope	−0.17	−0.27, −0.06	0.0035	−0.11	−0.14, −0.08	<0.001	−0.08	−0.10, −0.06	<0.001	−0.25	−0.27, −0.23	<0.001
Level change (immediate effect)	4.66	3.79, 5.53	<0.001	−0.33	−0.90, 0.23	0.243	2.24	1.60, 2.88	<0.001	1.58	1.07, 2.09	<0.001
Slope change (gradual effect)	−0.30	−0.51, −0.08	0.0089	0.04	−0.01, 0.10	0.127	0.06	0.02, 0.09	0.002	0.05	0.03, 0.08	<0.001
**Uncontrolled blood pressure**												
Pre-dementia slope	−0.0041	−0.0050, −0.0032	<0.001	−0.0086	−0.1514, −0.0021	0.011	–	–	–	–	–	–
Level change (immediate effect)	−0.11	−0.14, −0.08	<0.001	−0.44	−0.60, −0.28	<0.001	–	–	–	–	–	–
Slope change (gradual effect)	−0.00041	−0.00158, 0.00076	0.48	0.0049	−0.0057, 0.0155	0.357	–	–	–	–	–	–
**Healthcare resource utilization**												
Pre-dementia slope	0.14	0.02, 0.25	0.018	–	–	–	0.11	0.01, 0.20	0.029	–	–	–
Level change (immediate effect)	12.48	10.68, 14.28	<0.001	–	–	–	1.50	0.36, 2.64	0.011	–	–	–
Slope change (gradual effect)	−0.55	−0.74, −0.35	<0.001	–	–	–	−0.05	−0.23, 0.13	0.58	–	–	–

### Secondary outcome: UBP

**Fig. 3:**
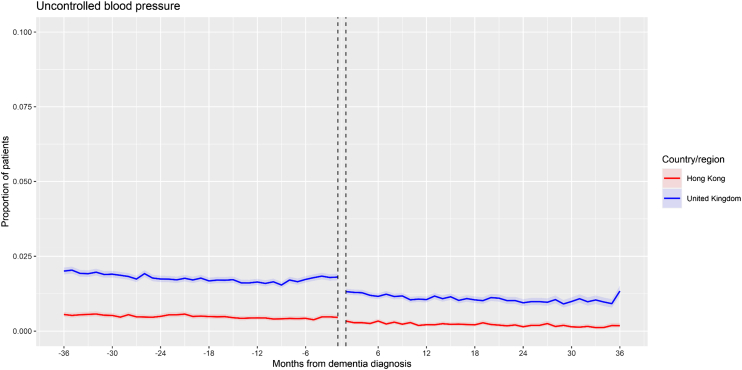
**Time series plot (Hong Kong, United Kingdom)—Uncontrolled blood pressure**. Note: Shaded areas represent 95% confidence intervals of monthly proportions.

Blood pressure measurements were only available in the Hong Kong and UK databases for the UBP outcome (Fig. 3). In both databases, a significantly negative pre-dementia slope was observed (decreasing trend in patients with UBP), followed by a significant decrease in level (immediate decrease in patients with UBP), followed by a non-significant slope change (continued decreasing trend). Overall, the proportions of patients with UBP were relatively low over the time series for both databases (<2.5%).

### Secondary outcome: HCRU

**Fig. 4:**
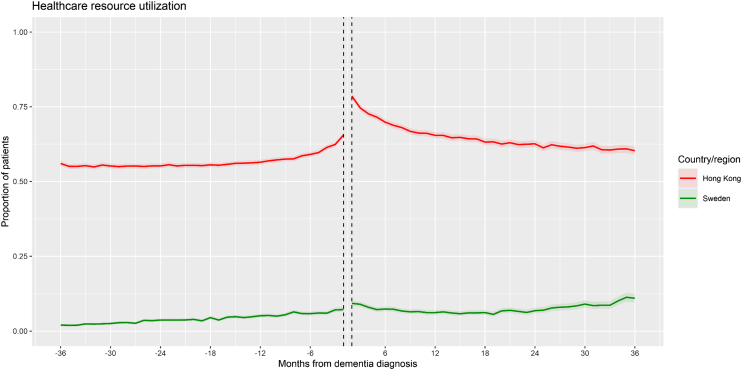
**Time series plot (Hong Kong, Sweden)—Healthcare resource utilization**. Note: Shaded areas represent 95% confidence intervals of monthly proportions.

Outpatient and inpatient records were only available in Hong Kong and Sweden for the HCRU outcome (Fig. 4). In both databases, a significantly positive pre-dementia slope was observed (increasing trend in patients with HCRU), followed by a significant increase in level (immediate increase in patients with HCRU). In Hong Kong, this was followed by a significant decrease in slope resulting in a negative post-dementia slope. In Sweden, the slope change was non-significant (continued increasing trend).

## Discussion

This is the largest population-based study to date using multiple databases investigating prescribing trends of BPLD in people living with dementia. In this study, prescribing patterns of antihypertensives before and after incident dementia diagnosis were described and evaluated in four databases in four countries/regions across three continents: Hong Kong, United Kingdom, Sweden, and Australia. In the time series analysis results, BPLD prescribing consistently decreased before dementia diagnosis across databases, increased immediately after dementia diagnosis in three out of four databases, and was followed by a decreasing trend with differing slope changes gradually after dementia diagnosis.

The increase in the proportion of patients with a BPLD prescription immediately after incident dementia diagnosis may suggest increased vigilance of blood pressure control and healthcare in general in the first few months after dementia diagnosis. As this is a common period where all patients were likely to receive prescriptions e.g. for other conditions or illnesses when concurrently receiving their diagnosis of dementia, this could have also contributed to the sharp level increase observed even after the removal of the three-month period around incident dementia diagnosis to mitigate this effect, although some surveillance bias may still remain. In the UK, this immediate increase in antihypertensive prescribing was not observed, which could be explained by the data being collected from primary care where patients may visit more frequently and fluctuations in prescriptions are less likely to occur.

The negative slopes observed in the slope change before and after incident dementia diagnosis indicates a decrease in the BPLD prescribing trend over the three years before and after dementia diagnosis. One possible explanation is that prescribing rates inadvertently receded over time after a period of heightened attention to blood pressure immediately after dementia diagnosis or patients simply forgetting to refill medications due to dementia. Changing healthcare practice in older adults diagnosed with dementia or with dementia symptoms are also likely and may explain the decreases in BPLD prescribing as long-term prevention of cardiovascular disease becomes less of a priority in these patients. The decreasing slope could also be attributed to deprescribing practices in older adults stemming from emergent deprescribing guidelines aimed to reduce polypharmacy burden.^31^^,^^32^ Furthermore, as there is evidence to suggest that hypertension in midlife is associated with an increased risk of dementia,^1^, ^2^, ^3^, ^4^ decreasing use of BPLD around the time of dementia diagnosis may not be such a concern with regards to the effect on dementia risk. Additionally, the decrease in BPLD prescribing was noticeably greater in the Hong Kong data. This could be due to greater severity of dementia as the mean age at dementia diagnosis was the highest in Hong Kong (81.1 years) suggesting relatively later diagnosis and a higher mortality rate being reported after dementia diagnosis in comparison with other countries.^33^ Finally, differences in healthcare system structures between the included countries/regions may also contribute to the differences in trends observed. Hong Kong and Australia have public and private healthcare systems as opposed to the universal healthcare systems in the UK and Sweden. As the data from Hong Kong only includes records from public hospitals, the greater decrease in prescribing observed in Hong Kong may be partially attributed to patients switching from public to private healthcare.

The results of the secondary outcomes, UBP and HCRU, also helped in part to explain the trend in antihypertensive prescribing. Although the overall proportion of patients with UBP were low, a negative slope with a significant immediate decrease in level after incident dementia diagnosis was observed, indicating that there were proportionately less patients with UBP over time. This suggests that less patients required antihypertensive prescriptions over time to control their blood pressure, which may reflect appropriate deprescribing and helps to explain the negative slope in BPLD prescribing. However, these results should be interpreted with caution due to the low overall proportions and the non-systematic nature of blood pressure measurements that can be dependent on indication for each patient. In the results for HCRU, the sharp level increase in BPLD immediately after incident dementia diagnosis was mirrored by a sharp level increase in HCRU, which implies that the increase in antihypertensive prescribing could be partly due to increased healthcare vigilance in the early months after dementia diagnosis.

Optimal management of hypertension in people living with dementia is not well described and evidence is scarce on how blood pressure treatment changes with regards to dementia diagnosis. This study builds on and adds to the existing literature on antihypertensive prescribing in dementia,^17^^,^^18^ describing BPLD prescribing trends before and after a diagnosis of dementia in four regions/countries. The findings of this study have both clinical and research implications. This study highlights how cardiovascular health, measured by BPLD prescribing, changes with dementia progression in those who eventually become diagnosed with dementia, and the subsequent trajectory of cardiovascular health management following a dementia diagnosis. Although a general immediate increase in prescribing was observed, there are many other considerations other than a history of hypertension when it comes to optimal blood pressure management in people living with dementia. These considerations include patients’ comorbidities (usually multiple and complex), the response in blood pressure measurements, and whether hypertension remains as a healthcare priority after a dementia diagnosis, which is usually made in older patients towards their end of life. Future studies can build on the method applied in this study and consider how datasets can be adapted to answer similar research questions involving trends before and after a healthcare event. Data that includes reasoning or indications for starting or stopping medication can also be extremely helpful in understanding how and why changes in trends might be observed.

One of the main strengths of this study is the large overall population size and ethnic diversity due to the inclusion of electronic health data from four regions/countries across three continents. The prescribing patterns observed can also be directly compared with each other as a common protocol approach was used to unify the study design across the collaborating sites. In addition, the time series analysis was a novel adaptation of the interrupted time series study design to investigate the impact of incident dementia diagnosis on BPLD prescribing trends, and this study provides a basis for further developments of this approach for similar research questions in the future. The three-year periods studied before and after incident dementia diagnosis is also a novel approach and was based on the median survival time of patients after being diagnosed with dementia.^22^ The validity and generalisability of this timeframe should be further evaluated in future research.

This study is also subject to limitations due to the differences in healthcare systems and the nature of data collection in the multiple databases used, which could have hampered the comparability of results. Some examples include: a large proportion of missing blood pressure measurement data in Hong Kong, loss of follow-up when transfer out of general practitioner practices in the UK, difficulty to detect healthcare utilization due to most patients with dementia being admitted to nursing homes with inhouse healthcare services in Sweden, and less precise dates of dementia diagnosis and death dates in the Australian database. However, the development of the common protocol aided in addressing these database differences and ensuring that the study could be conducted in a similar fashion across databases. Another limitation is that variation in follow-up might influence the observed prescribing trends, particularly in the post-diagnosis period, by inducing selection bias as patients included towards the tail end of the period may not be representative of the original population. However, patients were only counted in the denominator for monthly proportions up until any loss of follow-up to ensure consistent interpretation. The proportions of patients without a blood pressure measurement throughout the study period was 34% and 17% in Hong Kong and the UK, respectively, with higher proportions on a monthly basis. Data with blood pressure more closely monitored are recommended to investigate how prescribing trends change in response to uncontrolled blood pressure readings in future studies. Potential confounding covariates were not controlled for in the analyses, which may have affected the accuracy of the estimates of the trends over the time period. However, covariates adjustment may not have been appropriate as the focus of the study was on describing real-world, population-level medication utilization patterns rather than estimating causal effects between exposures and outcomes. Many of the estimates were also statistically significant due to the large sample size and potential for surveillance bias during the period of increased attention to healthcare around the time of dementia diagnosis, even when effect sizes may be trivial. The practical significance of the results was determined based on both visual interpretation of the time series along with the estimates. In the statistical analysis, a relatively simple autoregressive linear model was used and possible nonlinear effects were not tested, which may have again affected the accuracy of the estimates. However, the directions of the trends and study conclusions are expected to remain the same even with the simpler linear models. Investigating medication prescription and collection as separate outcomes may have been useful to identify changes in prescribing or patient behaviour around the time of dementia diagnosis, but separating these out in the prescription records among the databases was not possible. In addition, not all databases could contribute to the results for secondary outcomes in the time series analysis due to data that were unavailable or insufficient for the analysis. Measurement limitations remain in this study, including the reliance on prescribing or dispensing data, the use of diagnosis codes as proxies for hypertension and dementia, and missing blood pressure measurements. Finally, the interpretability of the study may be complicated by the multifactorial underlying mechanisms of the observed trends that cannot be distinguished from electronic health records, which may include a mixture of patient behaviour, prescriber decision-making, and differences in healthcare systems between countries/regions.

In conclusion, this population-based multinational database study described and compared antihypertensive prescribing trends in four regions/countries: Hong Kong, United Kingdom, Sweden, and Australia. The trend before and after incident dementia diagnosis was evaluated in a time series analysis. This study provided evidence on the general use of blood pressure-lowering drugs among people before and after a diagnosis of dementia and the results will be relevant to clinical practice in both local and global settings where ageing is a major public health concern.

## Contributors

ECLC and CSLC were responsible for the study concept, development, and design. ECLC, YW, LKE, and MA directly accessed and verified the data, collected data from each of their respective source databases and conducted the statistical analysis. ECLC wrote the original draft of the manuscript. MS, SS, NP, RB, YHJ, RDS, HL, JKY, and SH provided critical input to the study analyses, design, and discussion. All authors contributed to the interpretation of the analysis, critically reviewed and edited the intellectual content, and were involved in the final approval of the manuscript.

## Data sharing statement

Data will not be made available to others as the data custodians have not given permission.

## Declaration of interests

CSLC has received grants from the Food and Health Bureau of the Hong Kong Government, Hong Kong Research Grant Council, Hong Kong Innovation and Technology Commission, Pfizer, IQVIA, MSD, and Amgen; personal fees from MSD and Primevigilance Ltd.; outside the submitted work, and is a non-executive director of Advance Data Analytics for Medical Science (ADAMS) Limited (HK). MS has received consulting fees from Pfizer, unrelated to the present study.
