# Supplementary material for: Prescribing trends and time series analysis of blood pressure-lowering drugs among patients with dementia: a multinational database study
Source: eClinicalMedicine. 2025 Oct 30;90:103595. doi: 10.1016/j.eclinm.2025.103595 (PMC12613062; doi:10.1016/j.eclinm.2025.103595)
Supplement: Supplementary Material [file mmc1.docx]

**Supplementary Material 1. Data source descriptions**

*Hong Kong – Clinical Data Analysis and Reporting System*

Clinical Data Analysis and Reporting System (CDARS) is a database developed by the Hong Kong Hospital Authority, a statutory body that manages Hong Kong’s public healthcare services. Healthcare services provided by Hospital Authority including primary, secondary and tertiary services are available to all HK residents and data is captured into electronic patient records. This database has been frequently used in previous studies.^1-3^ These contain patients’ data including basic demographic information, diagnoses, prescription information, laboratory tests, admission and discharge information that is entered by trained hospital staff. There are also a multitude of data warehouses including data from the accident and emergency department, medical record system, in-patient, out-patient, and the pharmacy. A unique patient reference number is generated by CDARS for each individual patient to facilitate data retrieval and further analysis.

*United Kingdom – IQVIA Medical Research Database/The Health Improvement Network*

The IQVIA Medical Research Database (IMRD) incorporating data supplied by The Health Improvement Network (THIN) database, is a propriety database of Cegedim SA. This database contains pseudonymised electronic primary care data from over 16 million patients in the UK, representing approximately 6% of the population.^4^ The validity of the IMRD-UK database (previously known as THIN) for research has been demonstrated in several studies.^5-7^ including the generalisability and study specific accuracy of dementia diagnoses.^8^ Data within the database includes demographic information, lifestyle (e.g., smoking and alcohol consumption), laboratory results, medical diagnoses and prescribing information.

*Sweden - Swedish Prescribed Drug Register and the Swedish National Patient Registry*

The Sweden Prescribed Drug Register (SPDR) is administrated by the Swedish National Board of Health and Welfare that has been used extensively for research.^9, 10^ It contains information on all drugs dispensed in Swedish pharmacies. The date of dispensation, drug classification in Anatomical Therapeutic Chemical code (ATC code), number of defined daily doses, and prescribed amount are recorded. The Swedish Dementia Registry (SveDem) is a national registry for patients with dementia.^11^ Patients are registered by the date when they were diagnosed with dementia. It contains social security number, data on demographics, body mass index, type of dementia, day or home care, date of death and other relevant information. A unique personal identification number was used to link the data from these two databases for the purpose of conducting this study.

*Australia – Pharmaceutical Benefits Scheme data collection*

The Pharmaceutical Benefits Scheme (PBS) 10% sample data collection^12^ is a database managed by the Department of Health in Australia. It contains information on prescriptions that qualify for the PBS subsidy program under the National Health Act 1953 for which a dispensing claim has been processed. Information available to researchers include medicine details (name, strength, formulation, quantity supplied), date of prescription, date of dispensing, deidentified patient demographics regarding gender, year of birth and year of death, prescriber and pharmacy information. The PBS 10% sample provides a unit-record format that is available by request for research, containing standardised and longitudinal dispensing records for a random 10% of all available individuals in Australia. The Rx-Risk Comorbidity Index was used to identify patients’ comorbidities from prescriptions as proxies for each condition using ATC codes.^13^ Dementia symptomatic treatment (i.e. dispensing of risperidone for the behavioural and psychological symptoms of dementia, donepezil, rivastigmine, galantamine or memantine) was used to define incident dementia, with the date of the first claim for any of these medicines defined as the date of dementia diagnosis. The type of dementia could not be differentiated in this dataset.

**Supplementary Material 2. Cohort characteristics of each database**

| **Database** | **Site** | **Database size (number of patients)** | **Ethnicity** | **Cohort entry period** | **Definition of incident dementia diagnosis** |
| --- | --- | --- | --- | --- | --- |
| Clinical Data Analysis and Reporting System (CDARS) | Hong Kong | 11 million | Chinese | 2005-2019 | First diagnosis of dementia within cohort entry period |
| IQVIA Medical Research Database | United Kingdom | 16 million | Ethnically diverse (majority identify as Caucasian) | 2000-2020 | First diagnosis of dementia within cohort entry period |
| Swedish Prescribed Drug Register (SPDR) linked to the Swedish Dementia Registry (SveDem) | Sweden | 2.6 million individuals in SPDR (>65 years) linked to 90,000 dementia cases | European ancestry | 2005-2019 | First diagnosis of dementia within cohort entry period |
| Pharmaceutical Benefits Scheme (PBS) data collection | Australia | Over 26 million citizens in Australia, approximately 2.6 million included in the 10% sample | Ethnically diverse (majority identify as Caucasian) | 2015-2020 | First dispensing of dementia symptomatic treatment within cohort entry period |

**Supplementary Material 3. Diagnosis code mapping for dementia subtypes and hypertension between databases**

|  | **Hong Kong** | **United Kingdom** | **Sweden** |
| --- | --- | --- | --- |
| **Dementia subtypes** | **ICD-9** | **Read code** | **ICD-10** |
| Alzheimer’s disease | 331.0 | F110.00, Eu00.00, Eu00z11, Eu00112, F110000, Eu00011, Eu00z00, Eu00200, F110100, Eu00100, Eu00113, Eu00111, Eu00000, Fyu3000, Eu00012, Eu00013 | G30.0, G30.1, G30.8, G30.9 |
| Vascular dementia | 290.40, 290.41, 290.42, 290.43 | Eu01.00, Eu01200, Eu01z00, Eu01300, Eu01000, Eu01y00 | F01.50, F01.51 |
| Unspecified dementia | 294.2, 294.8, 290.0, 290.1, 290.2, 290.3, 290.8, 290.9 | Eu02z00 | F03.90, F03.91 |
| Other causes | 294.1, 291.2, 292.82 | E00..12, E00..11, Eu02z14, 1461, E000.00, E004.11, Eu02300, Eu01.11, F111.00, Eu01100, Eu02.00, E001.00, E002000, E004.00, E002100, E041.00, Eu10711, E012.11, E001300, Eu02z16, E001200, E00..00, Eu02z13, Eu02200, E003.00, E001z00, E002z00, Eu02400, E004z00, E001000, E004000, E004300, E002.00, Eu02z11, E001100, Eu04100, Eu02100, E012.00, E004200, Eu01111, E004100, Eu02y00 | F02.80, F02.81 |
| Frontotemporal dementia | 331.1 | Eu02000 | G31.0 |
| Dementia with Lewy bodies | 331.82 | F116.00, Eu02500 | G31.83 |
| **Hypertension** | **ICD-9** | **Read code** | **ICD-10** |
|  | 401-405 | 14A2.00, 1JD..00, 662..12, 6627.00, 6628.00, 6629.00, 662F.00, 662G.00, 662O.00, 662P.00, 662P000, 662b.00, 662c.00, 662d.00, 662q.00, 67H8.00, 7Q01.00, 7Q01y00, 8B26.00, 8BL0.00, 8CR4.00, 8HT5.00, 8I3N.00, 9N03.00, 9N1y200, 9OI..00, 9OI..11, F421300, G2...00, G2...11, G20..00, G20..11, G20..12, G200.00, G201.00, G202.00, G203.00, G20z.00, G20z.11, G21..00, G210.00, G210000, G210100, G210z00, G211.00, G211000, G211100, G211z00, G21z.00, G21z000, G21z011, G21z100, G21zz00, G22..00, G220.00, G221.00, G222.00, G22z.00, G22z.11, G23..00, G230.00, G231.00, G232.00, G233.00, G234.00, G23z.00, G24..00, G240.00, G240000, G240z00, G241.00, G241000, G241z00, G244.00, G24z.00, G24z000, G24z100, G24zz00, G25..00, G25..11, G250.00, G251.00, G26..00, G26..11, G27..00, G28..00, G2y..00, G2z..00, G672.00, G672.11, G8y3.00, Gyu2.00, Gyu2000, Gyu2100, L122.00, L122000, L122100, L122300, L122z00, L127.00, L127z00, L128.00, L128000, L128200, TJC7.00, TJC7z00, U60C500, U60C511, U60C51A | I10-I15 |

**Supplementary Material 4. ATC code and British National Formulary chapter mapping for blood pressure-lowering drugs/antihypertensives and anti-dementia drugs**

| **Drug class** | **ATC code** | **British National Formulary chapter** |
| --- | --- | --- |
| **Blood pressure-lowering drugs** |  |  |
| ACE inhibitors | C09A | 2.5.5.1 |
| Angiotensin II receptor blockers and other agents acting on renin-angiotensin system | C09C, C09X | 2.5.5.2, 2.5.5.3 |
| Calcium channel blockers | C08 | 2.6.2 |
| Diuretics | C03 | 2.2 |
| Beta-blockers | C07 | 2.4 |
| Vasodilators | C02 | 2.5.1 |
| Centrally acting alpha-2 agonists | C02 | 2.5.2 |
| Alpha-1 blockers | C02 | 2.5.4 |

**Supplementary Material 5. Time series analysis: monthly proportion of patients prescribed with an outcome of antihypertensive prescription, uncontrolled blood pressure or healthcare resource utilization**

Y_t_ = β_0_ + β_1_*Time1 + β_2_*Dementia + β_3_*Time_2_ + AR_n_ + ε_t_

- Y_t_: The proportion of patients prescribed with antihypertensives in month t
- Time_1_: Integer month order from up to 3 years before incident dementia diagnosis
- Time_2_: Integer month order from up to 3 years after incident dementia diagnosis
- Dementia: Dichotomous indicator for period after incident dementia diagnosis
- β_0_: Intercept
- β_1_: Slope before intervention
- β_2_: Level change after dementia period
- β_0_ + β_2_: Level after dementia period
- β_3_: Slope change after dementia period
- β_1_ + β_3_: Slope after dementia period
- AR_n_: Autoregressive terms at order n

Time series analysis can be classified into instrumental variable analysis using time as the instrument and is free from any confounding. Backwards stepwise autoregression will be used to determine the order n. ε_t_ is the stochastic error term at time t. The outcome measures will be the change in level and slope of the three outcomes: monthly proportion of patients prescribed with antihypertensives, monthly proportion of patients with uncontrolled blood pressure and monthly proportion of patients with a record of healthcare resource utilization. Durbin-Watson tests were used to detect autocorrelation.

**Supplementary Material 6. Charlson Comorbidity Index (CCI) Points^14^**

| **Characteristic/comorbidity** | **Points** |
| --- | --- |
| Age |  |
| <50 years | 0 |
| 50-59 years | 1 |
| 60-69 years | 2 |
| 70-79 years | 3 |
| ≥80 years | 4 |
| Myocardial infarction | 1 |
| Congestive heart failure | 1 |
| Peripheral vascular disease | 1 |
| Cerebrovascular disease | 1 |
| Chronic pulmonary disease | 1 |
| Connective tissue disease | 1 |
| Ulcer disease | 1 |
| Mild liver disease | 1 |
| Moderate/severe liver disease | 3 |
| Diabetes | 1 |
| Diabetes with end-organ damage | 2 |
| Hemiplegia/paraplegia | 2 |
| Moderate/severe renal disease | 2 |
| Any tumour | 2 |
| Metastatic solid tumour | 6 |
| Leukaemia | 2 |
| Lymphoma | 2 |
| AIDS | 6 |

**CCI = Sum of points for each individual**

REFERENCES

1. Wan EYF, Chui CSL, Lai FTT, Chan EWY, Li X, Yan VKC, et al. Bell's palsy following vaccination with mRNA (BNT162b2) and inactivated (CoronaVac) SARS-CoV-2 vaccines: a case series and nested case-control study. The Lancet Infectious diseases. 2022;22(1):64-72.

2. Lai FTT, Yan VKC, Ye X, Ma T, Qin X, Chui CSL, et al. Booster vaccination with inactivated whole-virus or mRNA vaccines and COVID-19-related deaths among people with multimorbidity: a cohort study. CMAJ : Canadian Medical Association journal = journal de l'Association medicale canadienne. 2023;195(4):E143-e52.

3. Huang C, Wei Y, Yan VKC, Ye X, Kang W, Yiu HHE, et al. Vaccine effectiveness of BNT162b2 and CoronaVac against SARS-CoV-2 omicron infection and related hospital admission among people with substance use disorder in Hong Kong: a matched case-control study. The lancet Psychiatry. 2023;10(6):403-13.

4. The Health Improvement Network (THIN) [Available from: <https://www.the-health-improvement-network.com/>]. Accessed on 16 October 2023.

5. Blak BT, Thompson M, Dattani H, Bourke A. Generalisability of The Health Improvement Network (THIN) database: demographics, chronic disease prevalence and mortality rates. Informatics in primary care. 2011;19(4):251-5.

6. Hall GC. Validation of death and suicide recording on the THIN UK primary care database. Pharmacoepidemiology and drug safety. 2009;18(2):120-31.

7. Lewis JD, Schinnar R, Bilker WB, Wang X, Strom BL. Validation studies of the health improvement network (THIN) database for pharmacoepidemiology research. Pharmacoepidemiology and drug safety. 2007;16(4):393-401.

8. McGuinness LA, Warren-Gash C, Moorhouse LR, Thomas SL. The validity of dementia diagnoses in routinely collected electronic health records in the United Kingdom: A systematic review. Pharmacoepidemiology and drug safety. 2019;28(2):244-55.

9. Wettermark B, Hammar N, Fored CM, Leimanis A, Otterblad Olausson P, Bergman U, et al. The new Swedish Prescribed Drug Register--opportunities for pharmacoepidemiological research and experience from the first six months. Pharmacoepidemiology and drug safety. 2007;16(7):726-35.

10. Wallerstedt SM, Wettermark B, Hoffmann M. The First Decade with the Swedish Prescribed Drug Register - A Systematic Review of the Output in the Scientific Literature. Basic & clinical pharmacology & toxicology. 2016;119(5):464-9.

11. Religa D, Fereshtehnejad SM, Cermakova P, Edlund AK, Garcia-Ptacek S, Granqvist N, et al. SveDem, the Swedish Dementia Registry - a tool for improving the quality of diagnostics, treatment and care of dementia patients in clinical practice. PloS one. 2015;10(2):e0116538.

12. Mellish L, Karanges EA, Litchfield MJ, Schaffer AL, Blanch B, Daniels BJ, et al. The Australian Pharmaceutical Benefits Scheme data collection: a practical guide for researchers. BMC research notes. 2015;8:634.

13. Pratt NL, Kerr M, Barratt JD, Kemp-Casey A, Kalisch Ellett LM, Ramsay E, et al. The validity of the Rx-Risk Comorbidity Index using medicines mapped to the Anatomical Therapeutic Chemical (ATC) Classification System. BMJ open. 2018;8(4):e021122.

14. Charlson Comorbidity Index (CCI) [Available from: <https://www.mdcalc.com/charlson-comorbidity-index-cci/>]. Accessed on 16 October 2023.
